# An immunologically relevant rodent model demonstrates safety of therapy using a tumour‐specific IgE

**DOI:** 10.1111/all.13455

**Published:** 2018-10-08

**Authors:** D. H. Josephs, M. Nakamura, H. J. Bax, T. S. Dodev, G. Muirhead, L. Saul, P. Karagiannis, K. M. Ilieva, S. Crescioli, P. Gazinska, N. Woodman, C. Lombardelli, S. Kareemaghay, C. Selkirk, H. Lentfer, C. Barton, S. Canevari, M. Figini, N. Downes, D. Dombrowicz, C. J. Corrigan, F. O. Nestle, P. S. Jones, H. J. Gould, P. J. Blower, S. Tsoka, J. F. Spicer, S. N. Karagiannis

**Affiliations:** ^1^ St. John's Institute of Dermatology School of Basic & Medical Biosciences King's College London London UK; ^2^ School of Cancer & Pharmaceutical Sciences Guy's Hospital King's College London London UK; ^3^ Randall Centre for Cell and Molecular Biophysics King's College London London UK; ^4^ Department of Informatics Faculty of Natural and Mathematical Sciences King's College London London UK; ^5^ Breast Cancer Now Research Unit School of Cancer & Pharmaceutical Sciences Guy's Cancer Centre King's College London London UK; ^6^ King's Health Partners Cancer Biobank School of Cancer & Pharmaceutical Sciences King's College London London UK; ^7^ Biotherapeutics Development Unit Cancer Research UK South Mimms UK; ^8^ Centre for Drug Development Cancer Research UK London UK; ^9^ Department of Applied Research and Technology Development Fondazione IRCCS Istituto Nazionale dei Tumouri Milan Italy; ^10^ Sequani Ledbury UK; ^11^ CHU Lille Institut Pasteur de Lille Inserm Univ. Lille Lille France; ^12^ Medical Research Council & Asthma UK Centre in Allergic Mechanisms of Asthma King's College London London UK; ^13^ Immunology and Inflammation Therapeutic Research Area Sanofi US Cambridge MA USA; ^14^ Imaging Chemistry & Biology Division of Imaging Sciences and Biomedical Engineering St. Thomas's Hospital King's College London London UK

**Keywords:** AllergoOncology, cancer, IgE, immunotherapy, rat

## Abstract

**Background:**

Designing biologically informative models for assessing the safety of novel agents, especially for cancer immunotherapy, carries substantial challenges. The choice of an in vivo system for studies on IgE antibodies represents a major impediment to their clinical translation, especially with respect to class‐specific immunological functions and safety. Fcε receptor expression and structure are different in humans and mice, so that the murine system is not informative when studying human IgE biology. By contrast, FcεRI expression and cellular distribution in rats mirror that of humans.

**Methods:**

We are developing MOv18 IgE, a human chimeric antibody recognizing the tumour‐associated antigen folate receptor alpha. We created an immunologically congruent surrogate rat model likely to recapitulate human IgE‐FcεR interactions and engineered a surrogate rat IgE equivalent to MOv18. Employing this model, we examined in vivo safety and efficacy of antitumour IgE antibodies.

**Results:**

In immunocompetent rats, rodent IgE restricted growth of syngeneic tumours in the absence of clinical, histopathological or metabolic signs associated with obvious toxicity. No physiological or immunological evidence of a “cytokine storm” or allergic response was seen, even at 50 mg/kg weekly doses. IgE treatment was associated with elevated serum concentrations of TNFα, a mediator previously linked with IgE‐mediated antitumour and antiparasitic functions, alongside evidence of substantially elevated tumoural immune cell infiltration and immunological pathway activation in tumour‐bearing lungs.

**Conclusion:**

Our findings indicate safety of MOv18 IgE, in conjunction with efficacy and immune activation, supporting the translation of this therapeutic approach to the clinical arena.

## 1 INTRODUCTION

Preclinical safety assessments for monoclonal antibodies (mAbs), especially novel immunomodulatory agents, attracted regulatory attention following the significant unexpected adverse events observed with an anti‐CD28 super‐agonist mAb targeting regulatory T cells during its first‐in‐human (FIH) clinical trial in 2006.^1^, ^2^ Identification of a pharmacologically relevant animal species for conducting safety studies is now incorporated in the European Medicines Agency guidance on taking novel therapeutics from preclinical studies into FIH clinical trials.^2^ Design of biologically and immunologically relevant models represents an even greater challenge for therapeutic agents with diverse, often multimodal functional profiles, or for those representing novel therapeutic classes.

For many therapeutic human IgGs, preclinical evaluation of Fc‐mediated immune functions is often performed in immunocompetent mice, because human IgG Fc interacts with murine FcγRs.^3^ For the same reason, preclinical safety evaluation of IgGs is conducted in mice or primates, to study the antibody intended for human administration. In contrast, mouse models for studies of IgE class‐specific immunological functions and safety, including for cancer immunotherapy, are not useful in the clinical translation of this class. Differences between species render the mouse system inadequately representative of human IgE biology. These include the absence of human IgE binding to murine FcεRs^4^, ^5^ and differential FcεRI structure (murine FcεRI is tetrameric, while human FcεRI exists both as a tetramer and a trimer).^4^ Murine FcεRIs are expressed solely on mast cells and basophils,^4^ whereas in rats, similar to humans, expression is on mast cells, basophils, eosinophils, monocytes and macrophages.^6^, ^7^ These features suggest the rat, rather than the mouse, as a model immune system better suited to preclinical evaluations of human IgE.

We previously reported the in vivo efficacy of recombinant chimeric (mouse/human) antibody, hMOv18 IgE, specific for the human tumour‐associated antigen folate receptor α (FRα), overexpressed in solid tumours including ovarian carcinomas. The antitumour activity of hMOv18 IgE was superior to the equivalent hMOv18 IgG in a syngeneic rat model, and in two human ovarian carcinoma xenograft mouse models reconstituted with human immune cells.^8^, ^9^, ^10^, ^11^, ^12^ In preparation for a clinical trial of hMOv18 IgE, we hypothesized that an immunocompetent rat model would provide the most comprehensive evaluation of safety and efficacy. This model was selected because it provides i) a species in which the native FcεR is expressed on effector cell populations similar to human cells, ii) an opportunity to study syngeneic tumours in highly vascularized lungs of immunocompetent animals, iii) a self‐replenishing supply of native effector cells and iv) a chance to evaluate antibody safety in the presence of antitumour IgE‐mediated responses in tumour‐bearing animals.^11^

Although human exposure to exogenous IgE has been reported,^13^ an IgE antibody recognizing a tumour antigen has not, until now, been introduced as a potential anticancer therapeutic. A conceivable reservation in contemplating a clinical trial is the perceived risk that intravenous IgE administration may trigger systemic or organ‐specific toxicities, including blood basophil activation and induction of inflammatory cascades, potentially leading to cytokine storm or allergic reactions. We previously explored the propensity of human antitumour IgE to trigger human blood basophil activation and mast cell degranulation in healthy volunteers’ and cancer patients’ blood ex vivo. We found no evidence of FcεRI‐mediated activation of basophils or mast cell degranulation with hMOv18 IgE.^14^ However, the safety of hMOv18 IgE in a physiologically and immunologically relevant immunocompetent tumour‐bearing in vivo system has not yet been evaluated.

Here, we report the design and implementation of a syngeneic tumour model in immunocompetent rats to examine IgE safety and compare antitumour efficacy with that of the equivalent IgG2b. Surrogate rat MOv18 IgE and IgG2b antibodies (rMOv18) were generated. Treated rats were subjected to clinical, histopathological and metabolic evaluation. Immune cell recruitment and immune mediator release were interrogated to provide preclinical insights into this most appropriate immunocompetent host.

## 2 METHODS

See Appendix S1 for details about cell lines, isolation of rat immune cells from blood, antibody binding to cell surface receptors, quantitative analysis of gene expression in antibody‐treated tumour‐bearing rat lungs, Gene Set Enrichment Analysis and multicytokine bead immunoassays.

### 2.1 Generation of anti‐FRα antibodies with human and rat Fc regions

**Figure 1:**
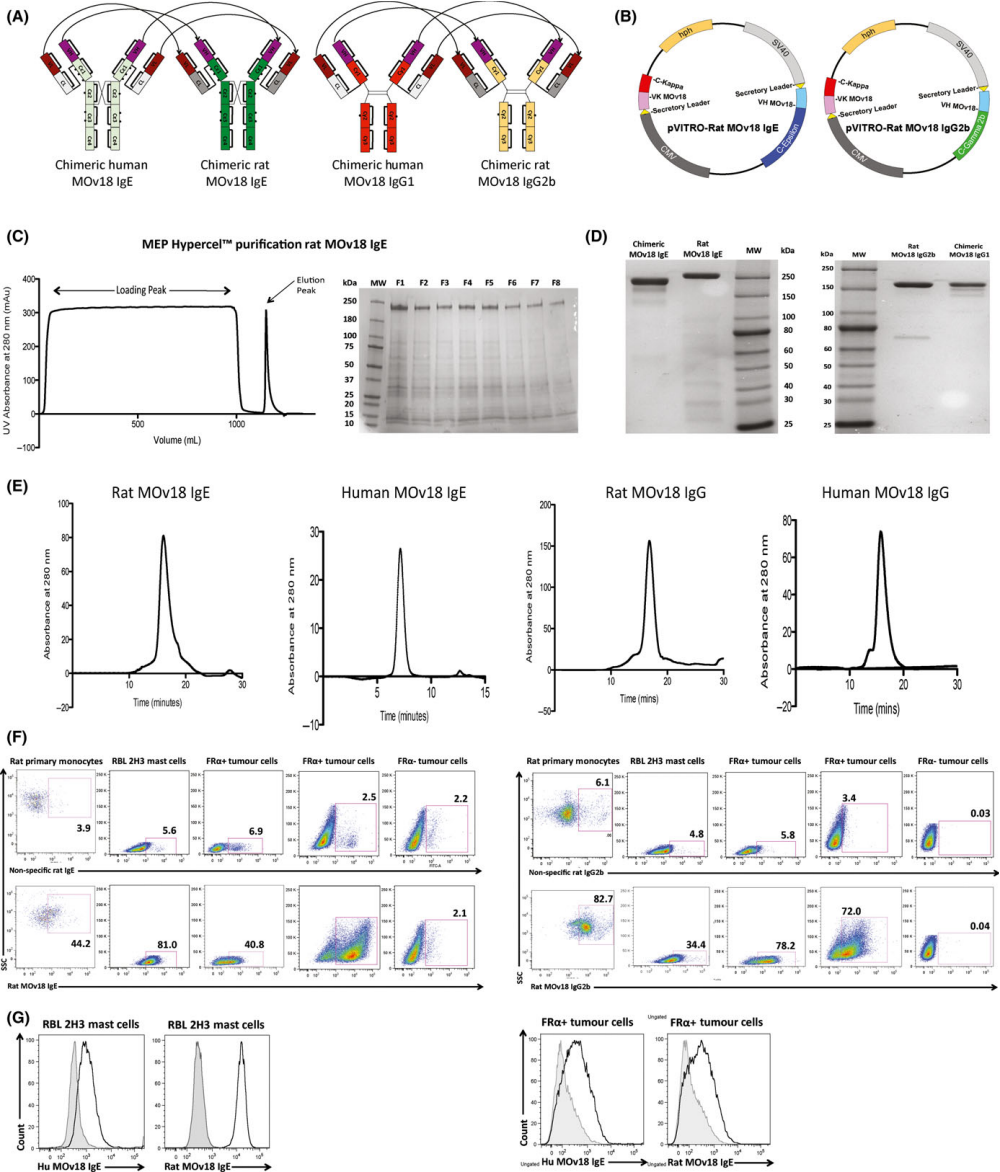
Rat MOv18 IgE and IgG2b antibody engineering, purification and characterization. A, Schematic representations of the cloned rat rMOv18 IgE and IgG2b chimeric antibodies with mouse variable domains specific for the human folate receptor α (FRα) and rat constant domains (light green: human Cε, dark green: rat Cε, dark orange: human Cγ, light orange: rat Cγ, light grey: human Cκ, dark grey: rat C_L_, purple: mouse V_H_, brown: mouse V_L_). B, The heavy and light chains of each rMOv18 antibody were cloned into a single mammalian expression vector per antibody (pVITRO1). This resulted in the production of pVITRO‐Rat MOv18 IgE and IgG2b. C, Purification of rMOv18 IgE with MEP HyperCel™ resin and 25% isopropanol elution buffer (MW: molecular weight marker, F1‐F8: fractions 1‐8). D, E, non‐reducing SDS‐PAGE (D) and HPLC‐SEC elution profiles (E) of HPLC‐purified hMOv18 IgE and IgG1, and rMOv18 IgE and IgG2b demonstrate intact monomeric antibodies of purities >85%. F, rMOv18 IgE and IgG2b bind to rat primary monocytes, RBL‐2H3 rat mast cells, FRα‐expressing rat colon adenocarcinoma CC531tFR cells and human ovarian carcinoma IGROV1 cells, but not to non‐FRα‐expressing human melanoma A375 cells. G, hMOv18 IgE shows weak binding, while rMOv18 IgE demonstrates significant binding to rat FcεRI‐expressing RBL‐2H3 cells. Human and rat MOv18 IgE display similar binding to human FRα‐expressing CC531tFR cells [Colour figure can be viewed at wileyonlinelibrary.com]

Chimeric mouse/human antibodies, hMOv18 IgE and IgG1 were prepared as before.^8^ Chimeric mouse/rat rMOv18 IgE and IgG2b were designed with rat constant and mouse variable domains specific for human FRα (Figure 1A). Heavy and light chain region sequences were cloned into single mammalian expression vectors containing a hygromycin resistance gene (pVITRO1)^15^ and transfected into FreeStyle™ 293‐F cells. Selected rMOv18 IgE‐ and IgG2b‐transfected cells were expanded using an AppliFlex Single‐Use Bioreactor (Applikon Biotechnology) (50L bioreactor wave bag, 25L working volume).

### 2.2 Purification of recombinant anti‐FRα antibodies with human and rat Fc regions

rMOv18 IgE and IgG2b antibodies were purified using MEP (4‐mercapto‐ethyl‐pyridine) HyperCel™ Mixed‐Mode Chromatography Sorbent (Pall International) and affinity chromatography using a HiTrap™ Protein G HP column (GE Healthcare), respectively.^16^ The MEP HyperCel™ resin was packed into an XK26/20 column (GE Healthcare) and equilibrated with PBS pH 7.0 (flow rate 5 mL/min). Supernatants were loaded onto columns, followed by washing with PBS pH 7.0. Captured rMOv18 IgE was eluted using 25% (v/v) isopropanol, 0.1 mol/L sodium chloride, 10 mmol/L phosphate (pH 7.0). Eluted antibodies were further purified by size exclusion chromatography (SEC) (Gilson HPLC, Superdex™ 200 10/300 GL column, GE Healthcare) and analysed by SDS‐PAGE chromatography. Antibody purity was calculated by comparing the area under the curve for individual peaks on the HPLC‐SEC chromatogram.

### 2.3 Assessments of antibodies in vivo

#### 2.3.1 Immunocompetent syngeneic WAG rat model of FRα‐expressing lung metastases

Female Wistar Albino Glaxo (WAG/RijCrl) rats (Charles River) were maintained and handled in accordance with the Institutional Committees on Animal Welfare of the UK Home Office in compliance with The Home Office Animals Scientific Procedures Act, 1986. Rats were injected intravenously with CC531tFR tumour cells and subsequently treated with rMOv18 antibodies on days 1 and 14 or on days 1, 7, 14 and 21. Animals were humanely euthanized, and lung tumour burden was determined by i) mean number of surface‐visible metastases/cm^2^ and ii) % tumour occupancy (total white surface area [mm^2^]/total lung (black + white) surface area [mm^2^]).^11^

#### 2.3.2 Clinical observations and measurements

Animals were examined twice daily for clinical signs of toxicity or changes in behaviour and appearance. On dosing days, animals were examined after dose completion and approximately 1 and 4 hours thereafter. Animals were weighed at the start of treatment and weekly thereafter. General toxicities were evaluated according to the Federation of European Laboratory Animal Science Association (FELASA) working group report on pain and distress in laboratory rodents^17^ (Table S1A). Signs of anaphylaxis were evaluated using a previously described anaphylactic scoring system^18^ (Table S1B).

#### 2.3.3 Clinical pathology

Blood samples were drawn into EDTA and lithium heparin collection tubes and analysed for haemoglobin concentration, total white blood cell count, neutrophil count, and creatinine, alanine aminotransferase and aspartate aminotransferase concentrations (haematological parameters: ADVIA 120 haematology analyser; biochemistry parameters: Roche Modular Evo (P800) clinical chemistry analyser). Full necropsies were performed at day 30 following tumour challenge. Tissues were preserved in neutral buffered formaldehyde before processing for histological evaluation.

### 2.4 Histological evaluations

Rat tissues (thymus, injection site (tail), spleen, lymph nodes, liver, kidney, lungs, colon and brain) were submerged in neutral buffered formaldehyde, embedded in paraffin wax, cut at a nominal thickness of 4‐5 μm, stained with haematoxylin and eosin and analysed on a Zeiss Axiophot microscope (×10, ×20 magnification lenses, Carl Zeiss) and NIS Elements imaging software (Nikon).

### 2.5 Statistical methods

Parametrically distributed data were compared with an unpaired two‐tailed Student's *t* test. Nonparametric data were compared using the Mann‐Whitney *U* test. All statistical analyses were performed using GraphPad™ Prism software (version 5.03, GraphPad™). *P* values were represented as follows: *P <* *.05, **P <* *.01, ***P <* *.001, ****P <* *.0001. Error bars represent SD and SEM in in vitro figures, and SEM in in vivo figures and histological analyses. All results represent at least 2 independent experiments.

## 3 RESULTS

### 3.1 Generation and receptor‐binding characteristics of rat MOv18 IgE and IgG2b

We generated anti‐human FRα mouse/rat chimeric (mouse variable/rat constant domains) rMOv18 IgE and IgG2b, surrogates of the original mouse/human chimeric hMOv18 IgE and IgG1, respectively. Rat IgG2b isotype was chosen as equivalent to mouse IgG2a/b and human IgG1, based on complement activation and antibody‐dependent cell‐mediated cytotoxicity (ADCC) functions (Figure 1A).^19^ Variable region DNA sequences for the MOv18 epitope on human FRα were combined with rat immunoglobulin constant region DNA sequences. The complete heavy and light chains of each rMOv18 IgE and IgG2b antibody (Figure 1A) were synthesized using GeneArt Gene Synthesis. Antibody chains were cloned within a dual antibody expression cassette into a single mammalian expression vector (pVITRO1), by employing an adapted and optimized Polymerase Incomplete Primer Extension (PIPE) cloning method that allowed seamless assembly of multiple DNA fragments.^15^ The pVITRO‐Rat MOv18 IgE and pVITRO‐Rat MOv18 IgG2b vectors **(**Figure 1B) contained a hygromycin B resistance gene, enabling antibiotic selection of transfected FreeStyle™ 293‐F cells. Semistable cell lines were generated for large‐scale antibody production.

As no universal purification method exists for rat IgE,^20^ we designed a strategy using a mixed‐mode chromatography resin, MEP HyperCel™, principally operating by hydrophobic charge induction chromatography (HCIC). The functional ligand, 4‐mercapto‐ethyl‐pyridine (MEP), is immunoglobulin‐selective and binds a broad range of immunoglobulin isotypes.^16^, ^21^ Adsorbed rMOv18 IgE was eluted using a 25% isopropanol elution buffer, and SDS‐PAGE revealed strong bands at the expected molecular weight (200 kDa for rat IgE)^22^ (Figure 1C). Following HPLC purification, human and rat MOv18 IgE (180 and 260 kDa) and MOv18 IgG (160 kDa) appeared as single bands,^22^ corresponding to expected antibody molecular weights (Figure 1D). HPLC‐SEC elution profiles confirmed the generation of monomeric antibodies of purity >85% (Figure 1E).

Purified rMOv18 IgE bound to rat FcεRI(αβγ2)‐expressing rat RBL‐2H3 mast cells and to primary rat peripheral blood monocytes (CD172 + ) expressing FcεRI(αγ2). The rMOv18 IgG2b bound primary rat monocytes (expressing FcγRI) and RBL‐2H3 cells (expressing low rFcγRII/FcγRIII levels). Both rat IgE and IgG2b bound to human FRα‐expressing, but not to FRα‐negative, tumour cells (Figure 1F). Consistent with previous findings,^7^ hMOv18 IgE showed weak binding, while rat IgE showed significant binding to rat FcεRI‐expressing RBL‐2H3 cells. Both human and rat MOv18 bound to human FRα‐expressing cancer cells (Figure 1G).

These findings confirm cloning and production of surrogate rMOv18 antibodies that recognize cognate Fc receptors on rat FcR+ immune cells and target antigen on FRα+ cancer cells.

### 3.2 Establishment of a tumour‐bearing surrogate rat model to interrogate IgE functions

**Figure 2:**
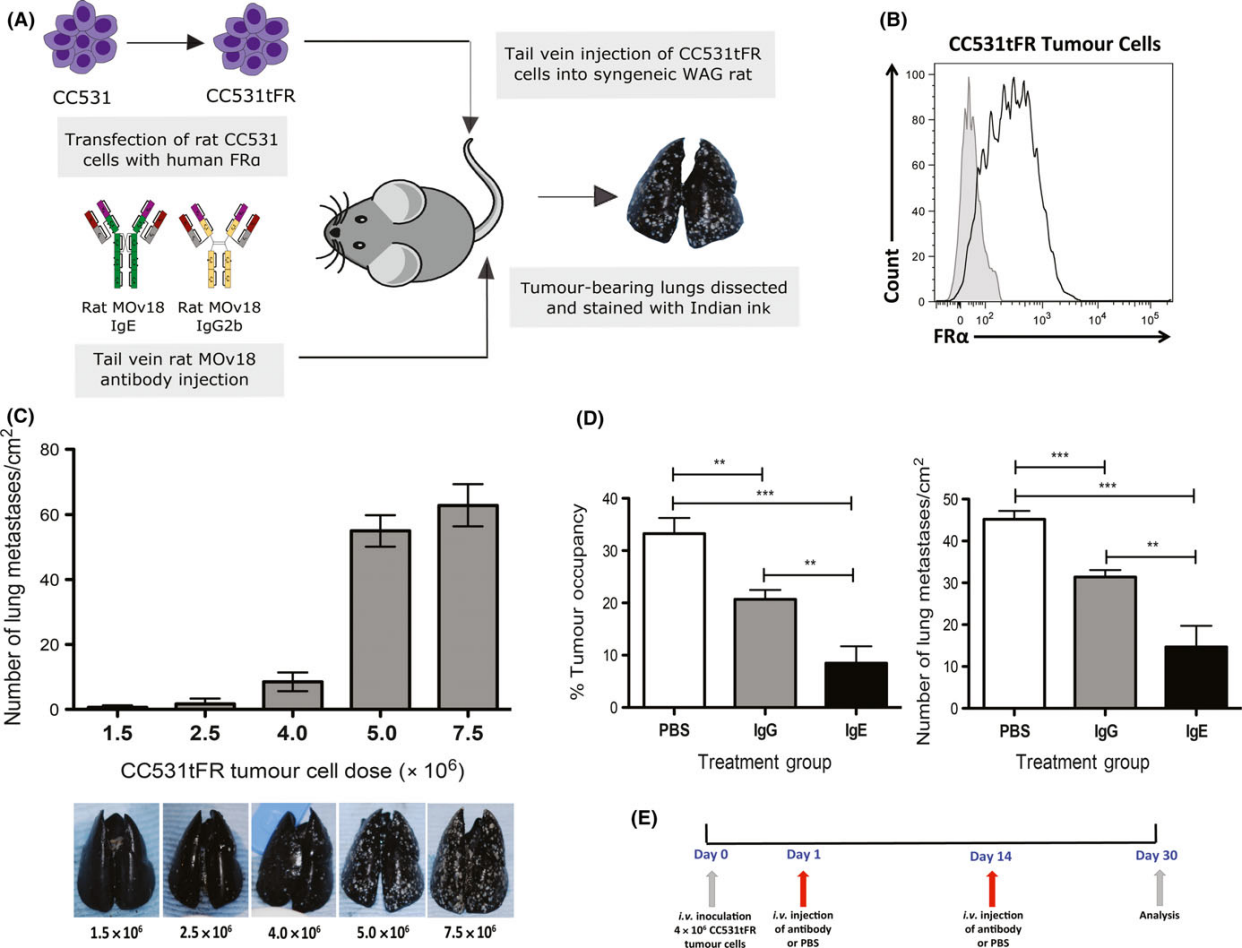
Establishment of the tumour‐bearing surrogate rat model and antibody‐mediated tumour growth restricting potencies. A, Schematic representation of immunocompetent model design: rMOv18 IgE engages native rat FcεR‐expressing immune effector cells to target syngeneic rat tumour cells expressing human folate receptor α (FRα). B, FRα expression of CC531tFR cells immediately prior to in vivo tumour challenge. C, Determination of optimum i.v. CC531tFR tumour challenge. Representative images of Indian ink‐stained lungs reveal white tumour lesions (n = 5). D, Rat MOv18 IgE demonstrates superior tumour growth restriction. Percentage (%) tumour occupancy and number of metastases/cm^2^ quantified following two doses (every 14 days) of PBS, or rMOv18 IgE and IgG2b at 10 mg/kg (n* = *6). rMOv18 IgE compared with rMOv18 IgG2b (mean % tumour occupancy: 8.5% vs 20.7%, *P *=* *.003; mean number of lung metastases/cm^2^: 14.7 vs 31.4, *P *=* *.0049) or with PBS (mean % tumour occupancy: 33.3%, *P *<* *.0001; mean number of lung metastases/cm^2^: 45.2, *P *<* *.0001). E, Dosing regimen following tumour challenge was on days 1 and 14 over a 30‐day period [Colour figure can be viewed at wileyonlinelibrary.com]

Folate receptor α expression is reported to be absent or lowly expressed in normal tissues,^23^ and the MOv18 clone may not cross‐react with the rat FRα homologue. We therefore designed a tumour model in immunocompetent WAG rats with which to study IgE Fc‐mediated antibody functions and safety. The syngeneic WAG rat adenocarcinoma cell line CC531 was transfected to express the human FRα (CC531tFR) and formed lung metastases following systemic challenge (Figure 2A). Flow cytometry confirmed expression of human FRα by CC531tFR cells (Figure 2B). At challenge doses between 1.5‐7.5 × 10^6^ tumour cells per rat, lung tumours developed in a dose‐dependent manner (number of lung metastases/cm^2^) (Figure 2C), and 4.0 × 10^6^ CC531tFR tumour cells per rat produced satisfactory, but not excessive, development of lung metastases. We administered antibodies at 2 doses (every 14 days) and confirmed significantly reduced lung metastases growth with 10 mg/kg rMOv18 IgE compared with rMOv18 IgG2b or PBS (Figure 2D‐E).

We thus confirmed the establishment of a tumour‐bearing surrogate rat model, in which we demonstrated restriction of syngeneic tumour growth by rat IgE and IgG2b antibodies, and showed enhanced efficacy of rMOv18 IgE compared with IgG2b.

### 3.3 Safety of rat MOv18 IgE and IgG2b in vivo

We interrogated the rat model with respect to safety using scoring criteria previously described to evaluate general toxicities in rodents (Table S1A) and in vivo anaphylactic responses (Table S1B).^17^, ^18^ We also conducted histopathological evaluations of several organs to detect tissue damage, especially from immunological infiltration or tissue remodelling.

**Figure 3:**
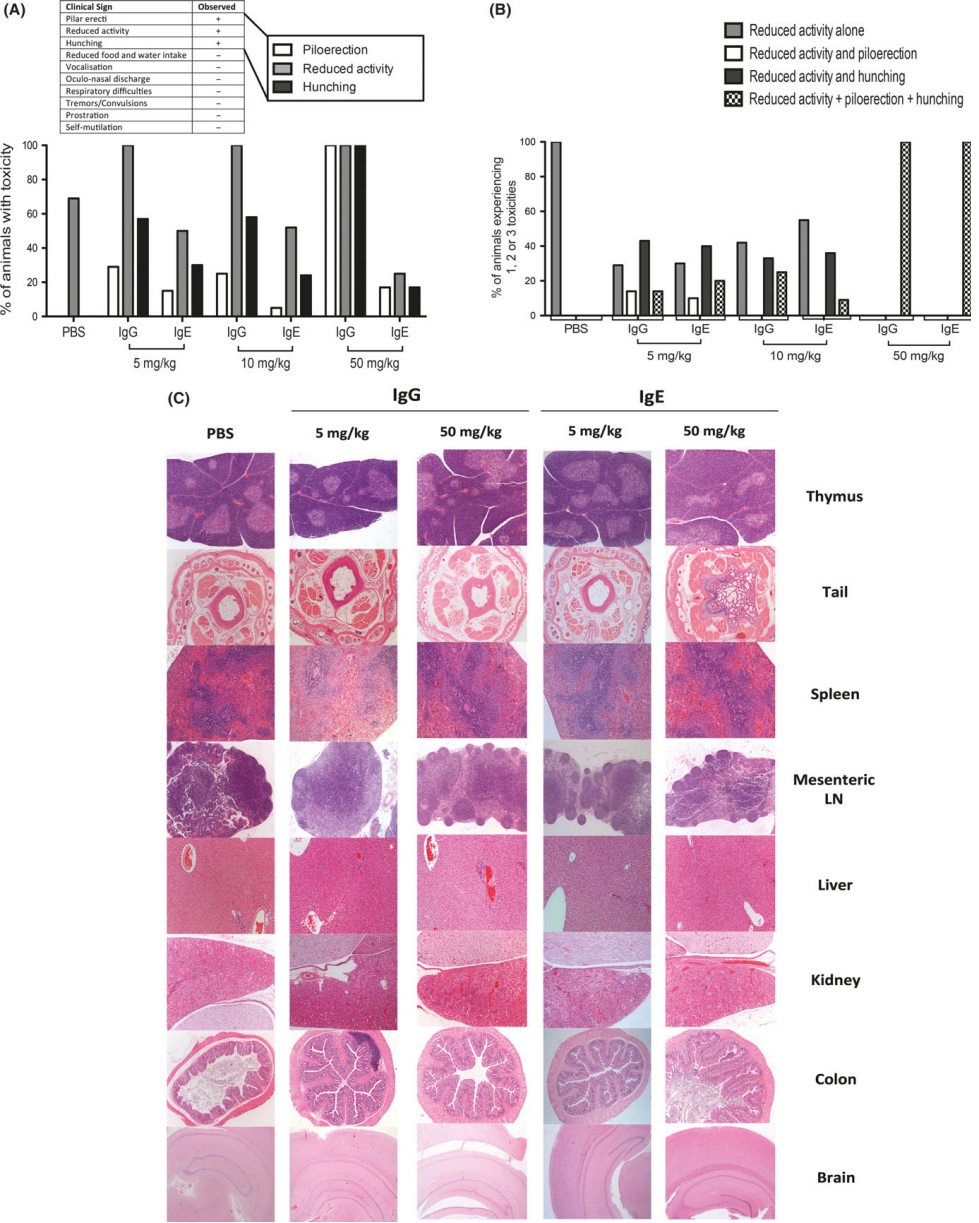
Rat MOv18 IgE and IgG induce comparable mild toxicities only. A, Percentage of rats in each treatment group experiencing the 3 detected toxicities (piloerection, hunching and reduced activity). B, Percentage of animals within each treatment group to experience 1, 2 or 3 toxicities. C, Histopathological evaluations of tissues 30 days after administration of two doses (every 14 days) of rMOv18 IgE or IgG2b at 5 mg/kg and 50 mg/kg or PBS rats (n* = *10 per group). Sections were stained with H&E and observed at brain = 25 × magnification; colon, kidney, liver, mesenteric lymph nodes (LN), tail and thymus = 50 × magnification; spleen = 100 × magnification [Colour figure can be viewed at wileyonlinelibrary.com]

Safety evaluations of rMOv18 IgE were conducted at weekly doses of 5, 10 and 50 mg/kg and compared with rMOv18 IgG2b and PBS. Buffer alone‐treated rats demonstrated a single toxicity of subdued but responsive behaviour (69%, mild score; Table S2). Of the possible general and anaphylactic toxicities, only 3 categories were observed in rats treated with rMOv18 IgE or IgG2b. These were as follows: subdued but responsive behaviour; piloerection; and hunching. All toxicities were scored as mild only, occurred immediately upon waking from anaesthetic, and lasted for 10‐15 minutes thereafter. Severity was similar for both IgE‐ and IgG2b‐treated rats (Table S2, Figure 3A). Additionally, the number of toxicities experienced by each animal in each treatment group was similar between the IgE‐ and IgG‐treated groups at each dose (Figure 3B). None of the rats in any treatment group experienced any symptom that would constitute an anaphylactic response, according to a published anaphylaxis scoring system (Table S1B).^18^

**Figure 4:**
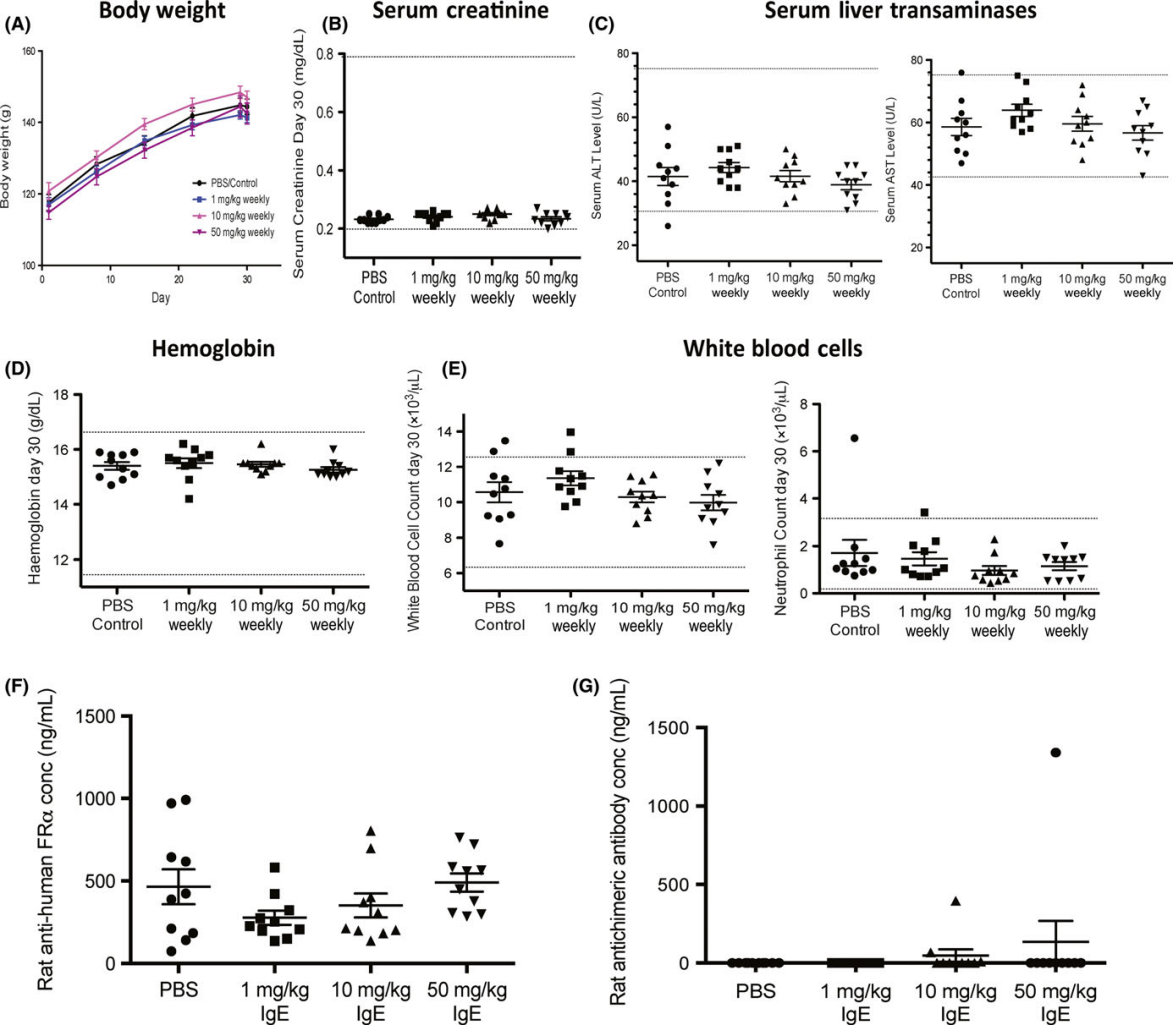
Absence of rMOv18 IgE‐associated changes in body weight or blood haematological or biochemical parameters. A, Weekly weights of rats following weekly treatment with rMOv18 IgE at 1 mg/kg (n = 10), 10 mg/kg (n = 10) and 50 mg/kg (n = 10) or PBS (n = 10). B‐E, Blood biochemical and haematological parameters were measured immediately before necropsy on day 30 following 4 weekly doses of rMOv18 IgE at 1 mg/kg (n = 10), 10 mg/kg (n = 10) and 50 mg/kg (n = 10) or PBS (n = 10). Dotted lines indicate normal range values for each parameter determined in healthy Wistar Albino Glaxo (WAG)/RijCrl.^24^ F‐G, Rat anti‐human folate receptor α (FRα) antibodies and rat antidrug antibodies (ADA) measured in rat serum following weekly treatment with PBS, or rMOv18 IgE at 1 mg/kg, 10 mg/kg and 50 mg/kg (n* = *10 per group) [Colour figure can be viewed at wileyonlinelibrary.com]

Histopathological evaluations of several tissues were conducted to determine potential off‐target toxicities. No obvious pathological lesions associated with 5 or 50 mg/kg IgE, or IgG2b administration, were detected in major physiological systems (spleen, lymph nodes, liver, kidney, colon, brain) (Figure 3C). IgE administration had no effect on body weight (Figure 4A), serum blood chemistry or haematological parameters (creatinine, liver transaminases, haemoglobin and white blood cell counts) compared with PBS treatment (Figure 4B‐E). As we previously reported in sera of patients with FRα‐expressing carcinomas,^14^ in this model, we found circulating antibodies recognizing FRα, irrespective of treatment or antibody dose. We detected antidrug antibodies (ADA) in the serum of one of ten rats treated with 10 mg/kg and in the serum of one of ten rats treated with 50 mg/kg IgE (Figure 4F‐G).

We concluded that administration of rMOv18 IgE to tumour‐bearing rats induced no clinical, histopathological or metabolic signs of a type I hypersensitivity reaction, even at weekly 50 mg/kg doses.

### 3.4 Immune cell infiltration and immune activation signatures associated with MOv18 IgE in vivo

**Figure 5:**
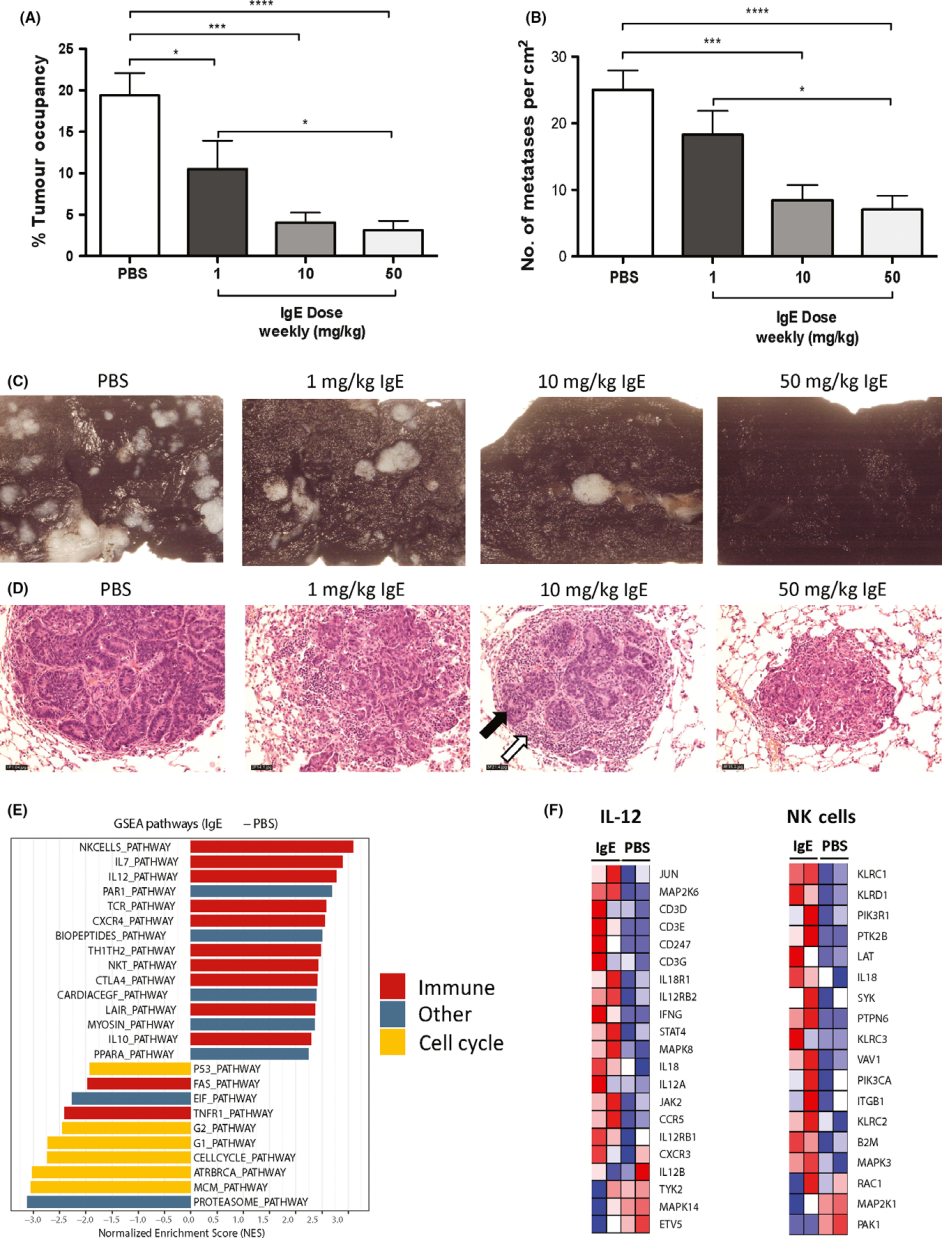
Rat MOv18 IgE induces immune cell infiltration and immune activating signatures. A, B, Percentage (%) tumour occupancy and number of metastases/cm^2^ quantified following four (weekly) doses of PBS, or rMOv18 IgE at 1 mg/kg, 10 mg/kg and 50 mg/kg (n* = *10 per group). C, Representative images of Indian ink‐stained lungs from PBS‐ and rMOv18 IgE‐treated rats (n* = *10 per group). D, Representative images of paraffin‐embedded lung sections from PBS‐ and rMOv18 IgE‐treated rats stained with H&E (n* = *10 per group). Magnification 200x. Black arrow: glandular tumour islet; white arrow: immune and stromal cells. E, GSEA analyses showing enrichment of immune‐associated signatures (red) in rMOv18 IgE‐treated (two doses, 2 weeks apart) tumour‐bearing rat lungs and cell cycle‐associated signatures (yellow) in PBS‐treated tumour‐bearing rat lungs. Other signatures are shown in blue. The *y*‐axis corresponds to the normalized enrichment score (NES). F, Heat maps showing the differential expression of all genes that constitute the IL‐12 and NK cells signature between rMOv18 IgE‐ and PBS‐treated rat lungs [Colour figure can be viewed at wileyonlinelibrary.com]

IgE antitumour efficacy is likely to function by recruitment of host immunity. We therefore investigated whether inhibition of lung metastases in IgE‐treated rats was associated with immune cell infiltration and activation. We measured growth inhibition of lung metastases with 4 (weekly) doses of rMOv18 IgE between 1, 10 and 50 mg/kg (Figure 5A‐C). Histological evaluation of rat lungs showed dose‐dependent reduction in tumour islet density, alongside immune and stromal cell infiltration appearing progressively greater with increasing doses of IgE compared with lesions from PBS‐treated rats (Figure 5D).

We generated mRNA expression profiles from tumour‐bearing rat lungs excised from animals treated with PBS or rMOv18 IgE. We employed Gene Set Enrichment Analysis (GSEA) to identify gene sets correlated with treatment. This yielded evidence of significant enrichment of gene expression associated with immune activation in IgE‐treated tumours, compared with enrichment of cell cycle‐associated pathways with vehicle treatment (Figure 5E). The IgE‐enhanced, activated genes included those associated with IL‐12 and NK cell immune activation pathways (Figure 5F).

**Figure 6:**
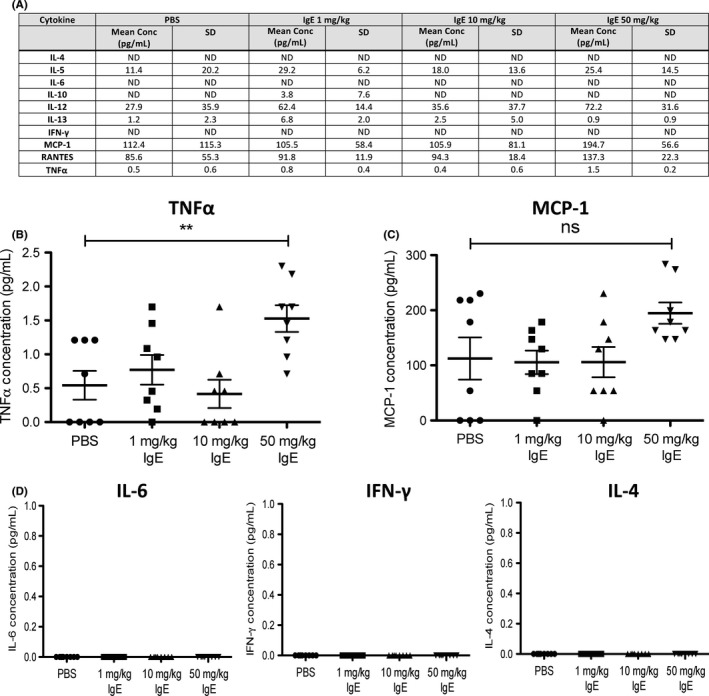
Rat MOv18 IgE triggers TNFα but no evidence of cytokine storm or allergic response. A, Cytokine/chemokine production measured in rat serum following weekly treatment with PBS, or rMOv18 IgE at 1 mg/kg, 10 mg/kg and 50 mg/kg (n* = *10 per group). B‐D, Concentrations of selected analytes in rat sera

We previously reported that TNFα‐expressing mature macrophage subsets infiltrated tumour lesions and that elevated levels of TNFα were detected in lungs of rMOv18 IgE‐treated rats compared with controls.^11^ Here, we also observed significantly elevated circulating TNFα in IgE‐treated compared with PBS‐treated rats (*P *=* *.0044) (Figure 6A‐B).^11^ Previously, we found elevated macrophage chemoattractant protein‐1 (MCP‐1) in the lung environment of IgE‐treated animals potentiated by local upregulation of TNFα following IgE cross‐linking on tumour‐associated macrophages. Here, we measured detectable serum MCP‐1 in IgE‐treated rats, but these were not significantly elevated compared with controls (Figure 6C). On the other hand, concentrations of the classical “cytokine storm”‐related cytokines IL‐6 and IFN‐γ were undetectable in rat sera from all groups. Furthermore, concentrations of IL‐4, a key cytokine in the development of allergic inflammation, were undetectable in sera of IgE‐treated and PBS‐treated rats (Figure 6D). These data are consistent with more prominent activation of immune cascades locally in tumours rather than systemically.

Together these findings point to immune cell recruitment, and activation of immunological pathways and mediators associated with IgE treatment, in the absence of any evidence of overt toxicities, or of any cytokine profiles that would indicate signs of cytokine storm or allergic response activation.

## 4 DISCUSSION

Identification of an appropriate animal model for studies involving IgE functions, including cancer immunotherapy, especially with regard to class‐specific immunological efficacy and safety, represents a major hurdle in clinical translation of this antibody class. Previous studies have reported substantial evidence of comparable or superior antitumour efficacy of hMOv18 IgE compared to its IgG counterpart.^8^, ^9^, ^10^, ^25^, ^26^ To translate this antitumour antibody safely into the clinic, studies conducted in a pharmacologically relevant species are required.

Here, we report findings in support of the in vivo safety of a tumour‐targeting IgE in an immunologically relevant immunocompetent syngeneic rat tumour model, selected because of similarities in the structure and distribution of FcεRI between humans and rats. We describe upregulation of immune‐associated pathways in lung metastases of IgE‐treated rats, associated with antitumour efficacy, and evidence of elevated serum concentrations of the pro‐inflammatory mediator TNFα. By contrast, neither the classical “cytokine storm”‐related cytokine IL‐6 nor the key allergic inflammatory cytokine, IL‐4, was upregulated. Despite the activation of immunological pathways associated with IgE treatment, no evidence of systemic or organ‐specific adverse events was detected.

We completed the rat model by generating surrogate rat IgE and IgG. We present a simple process for cloning heavy and light chains of any antibody clone into a single mammalian expression vector and report serum‐free production by mammalian cells by scale‐up in bioreactors. These processes are easily adaptable for generating antibodies of any isotype or specificity. Furthermore, the only currently reported purification method for monoclonal rat IgE is for a dinitrophenyl‐specific rat IgE (DNP IgE), purified by an affinity column based on antigen specificity.^20^ By contrast, our purification process for rat IgE regardless of antigen specificity using HCIC is also applicable for human IgE purification. Engineering, cloning and purification of intact, functional IgE of sufficient purity and yield for in vivo studies reported in the design of this rat model represents a methodology applicable to the generation of IgE and IgG antibodies of any specificity and species.

Administration of rMOv18 IgE and IgG2b to tumour‐bearing rats demonstrated only three toxicities, observed with both isotypes. Accordingly, none of the anaphylaxis‐specific toxicities detailed in previously published criteria^18^ were detected with any treatment. Moreover, immunohistochemical toxicity studies on several tissues from IgE‐, IgG2b‐ and PBS‐treated rats revealed no evidence of pathological lesions, even at weekly 50 mg/kg antibody doses. Together with our previously published data, these findings support clinical systemic MOv18 IgE administration.^14^

We previously reported that human IgE cross‐linked on the surface of human monocytes triggered monocyte activation and TNFα upregulation; furthermore, human IgE antitumour ADCC was associated with enhanced TNFα. In parallel, we detected TNFα‐expressing macrophage subsets and elevated concentrations of TNFα in lung metastases of IgE‐treated rats.^11^ Consistent with these, here IgE treatment enhanced immune cell recruitment in rat lung tumours, and activation of immunological pathways in tumour‐bearing lungs, alongside elevated TNFα in the sera of IgE‐treated rats compared with controls. Although previously we measured elevated concentrations of MCP‐1 alongside enhanced TNFα in rat bronchoalveolar lavage fluid of IgE‐treated animals, here, we did not observe statistically significant elevation of serum MCP‐1 in IgE‐treated rats. This may be because IgE Fc‐mediated cross‐linking on tumour‐associated macrophages can trigger elevated TNFα, stimulating local tumour cells to produce MCP‐1 in the tumour, and not systemically.^11^ It is possible that tissue‐resident mast cells may also contribute to IgE‐mediated enhanced TNFα expression, and this requires further investigation.^27^ These suggest principally local induction of tumour‐associated immune mediator release, immune cell activation and recruitment by IgE immunotherapy.

The immunocompetent syngeneic tumour model may also pose certain limitations, including lack of human FRα expression by normal rat tissues, and unlikely cross‐reactivity of rat FRα homologue with rat MOv18 IgE. Although observed in the circulation of a proportion of patients with FRα tumours such as ovarian carcinomas and mesotheliomas,^28^ we could not detect circulating shed human FRα (from CC531tFR cells) in rat sera in any of our studies (data not shown). Furthermore, FcεRI‐expressing cells in rats remain insufficiently characterized compared with human cells. Finally, laboratory rats, similar to mice, are not exposed to environmental allergens and thus may not display the array of allergen‐reactive antibodies potentially found in human patients. In the light of these, other species such as canine patients who spontaneously develop FRα‐expressing carcinomas may be interrogated. Considerable immunological homology between humans and dogs, alongside potential to better recapitulate the complexities of the microenvironment in spontaneously developed tumours in an immunocompetent species, may further benefit evaluations of antitumour IgE antibodies.^29^

In conclusion, we designed and implemented an immunologically relevant and physiologically representative tumour model which supports the preclinical safety of an antitumour IgE antibody, MOv18 IgE. Simultaneously, using this model, we report efficacy of the therapy in inducing tumour immune cell infiltration and immune pathway activation in the absence of physiological or immunological evidence of cytokine storm or allergic reaction. Our findings support the translation of IgE into the clinic and contribute to the rapidly growing field of AllergoOncology. Design of this and other immunologically congruent models may also help delineate IgE class‐specific functions in allergic inflammation, allergen immunotherapy and antiparasitic responses.

## CONFLICT OF INTERESTS

SNK and JFS are founders and shareholders of IGEM Therapeutics Ltd. FON is an employee of Sanofi US. All other authors declare no conflict of interests.

## AUTHOR CONTRIBUTIONS

D.H.J. and M.N. designed experiments. D.H.J., M.N., H.J.B., T.D., G.M., L.S., P.K., K.M.I., S.C., P.G., N.W., C.L., S.K., C.S., H.L., S.C., M.F. and N.D. performed the research. D.H.J., M.N., H.J.B., T.D., G.M., L.S., P.K., K.M.I., S.C., P.G., N.W., C.L., S.K., C.S., H.L., S.C., M.F., N.D., S.N.K. and J.F.S analysed the data. D.H.J., M.N., H.J.B., T.D., G.M., F.O.N., P.S.J., H.J.G., P.J.B., S.T., S.N.K. and J.F.S interpreted the data. D.H.J., H.J.B., T.D., G.M., F.O.N., P.S.J., H.J.G., P.J.B., S.T., S.N.K. and J.F.S wrote the manuscript. L.S., P.K., K.M.I., S.C., P.G., N.W., C.L., S.K., C.S., H.L., S.C., M.F. and N.D. all generated or provided key reagents. F.O.N., P.S.J., H.J.G., P.J.B. and S.T. discussed the data. S.N.K. and J.F.S conceived this study. S.N.K. designed the study, supervised and coordinated the project.

## References

[1] Suntharalingam G , Perry MR , Ward S , et al. Cytokine storm in a phase 1 trial of the anti‐CD28 monoclonal antibody TGN1412. N Engl J Med. 2006;355:1018‐1028. 16908486 10.1056/NEJMoa063842

[2] European Medicines Agency, Committee for Medicinal Products for Human Use (CHMP) . Guideline on strategies to identify and mitigate risks for first‐in‐human and early clinical trials with investigational medicinal products. 2017. 10.1111/bcp.13550 PMC6005602 29451320

[3] Dekkers G , Bentlage AEH , Stegmann TC , et al. Affinity of human IgG subclasses to mouse Fc gamma receptors. mAbs. 2017;9:767‐773. 28463043 10.1080/19420862.2017.1323159 PMC5524164

[4] Kinet JP . The high‐affinity IgE receptor (Fc epsilon RI): from physiology to pathology. Annu Rev Immunol. 1999;17:931‐972. 10358778 10.1146/annurev.immunol.17.1.931

[5] Bettler B , Hofstetter H , Rao M , Yokoyama WM , Kilchherr F , Conrad DH . Molecular structure and expression of the murine lymphocyte low‐affinity receptor for IgE (Fc epsilon RII). Proc Natl Acad Sci USA. 1989;86:7566‐7570. 2529542 10.1073/pnas.86.19.7566 PMC298106

[6] Dombrowicz D , Quatannens B , Papin JP , Capron A , Capron M . Expression of a functional Fc epsilon RI on rat eosinophils and macrophages. J Immunol. 2000;165:1266‐1271. 10903725 10.4049/jimmunol.165.3.1266

[7] Mallamaci MA , Chizzonite R , Griffin M , et al. Identification of sites on the human Fc epsilon RI alpha subunit which are involved in binding human and rat IgE. J Biol Chem. 1993;268:22076‐22083. 8408065

[8] Gould HJ , Mackay GA , Karagiannis SN , et al. Comparison of IgE and IgG antibody‐dependent cytotoxicity in vitro and in a SCID mouse xenograft model of ovarian carcinoma. Eur J Immunol. 1999;29:3527‐3537. 10556807 10.1002/(SICI)1521-4141(199911)29:11<3527::AID-IMMU3527>3.0.CO;2-5

[9] Karagiannis SN , Wang Q , East N , et al. Activity of human monocytes in IgE antibody‐dependent surveillance and killing of ovarian tumor cells. Eur J Immunol. 2003;33:1030‐1040. 12672069 10.1002/eji.200323185

[10] Karagiannis SN , Bracher MG , Hunt J , et al. IgE‐antibody‐dependent immunotherapy of solid tumors: cytotoxic and phagocytic mechanisms of eradication of ovarian cancer cells. J Immunol. 2007;179:2832‐2843. 17709497 10.4049/jimmunol.179.5.2832

[11] Josephs DH , Bax HJ , Dodev T , et al. Anti‐folate receptor‐alpha IgE but not IgG recruits macrophages to attack tumors via TNFalpha/MCP‐1 signaling. Cancer Res. 2017;77:1127‐1141. 28096174 10.1158/0008-5472.CAN-16-1829 PMC6173310

[12] Karagiannis SN , Josephs DH , Bax HJ , Spicer JF . Therapeutic IgE antibodies: harnessing a macrophage‐mediated immune surveillance mechanism against cancer. Cancer Res. 2017;77:2779‐2783. 28526770 10.1158/0008-5472.CAN-17-0428

[13] Dreskin SC , Goldsmith PK , Strober W , Zech LA , Gallin JI . Metabolism of immunoglobulin E in patients with markedly elevated serum immunoglobulin E levels. J Clin Investig. 1987;79:1764‐1772. 3584468 10.1172/JCI113017 PMC424519

[14] Rudman SM , Josephs DH , Cambrook H , et al. Harnessing engineered antibodies of the IgE class to combat malignancy: initial assessment of FcvarepsilonRI‐mediated basophil activation by a tumour‐specific IgE antibody to evaluate the risk of type I hypersensitivity. Clin Exp Allergy. 2011;41:1400‐1413. 21569129 10.1111/j.1365-2222.2011.03770.x

[15] Dodev TS , Karagiannis P , Gilbert AE , et al. A tool kit for rapid cloning and expression of recombinant antibodies. Sci Rep. 2014;4:5885. 25073855 10.1038/srep05885 PMC4115235

[16] Schwart W , Judd D , Wysocki M , Guerrier L , Birck‐Wilson E , Boschetti E . Comparison of hydrophobic charge induction chromatography with affinity chromatography on protein A for harvest and purification of antibodies. J Chromatogr A. 2001;908:251‐263. 11218128 10.1016/s0021-9673(00)01013-x

[17] FELASA . Pain and distress in laboratory rodents and lagomorphs. Report of the Federation of European Laboratory Animal Science Association (FELASA) Working Group on Pain and Distress. Lab Anim. 1994;28:97‐112. 8035572 10.1258/002367794780745308

[18] Li Z , Gao Y , Wang H , Liu Z . A rat model of Shuang Huang Lian injection‐induced anaphylaxis. Asian Pac J Allergy Immunol. 2010;28:185‐191. 21038789

[19] Bruggemann M , Williams GT , Bindon CI , et al. Comparison of the effector functions of human immunoglobulins using a matched set of chimeric antibodies. J Exp Med. 1987;166:1351‐1361. 3500259 10.1084/jem.166.5.1351 PMC2189658

[20] Hanashiro K , Nakamura M , Tamaki N , Kosugi T . Production of a monoclonal dinitrophenyl‐specific rat IgE and establishment of an IgE capture ELISA for estimating the concentration of rat IgE antibodies to dinitrophenyl‐Ascaris suum. Int Arch Allergy Immunol. 1996;110:371‐379. 8768805 10.1159/000237330

[21] Boschetti E . Antibody separation by hydrophobic charge induction chromatography. Trends Biotechnol. 2002;20:333‐337. 12127280 10.1016/s0167-7799(02)01980-7

[22] Bazin H , Querinjean P , Beckers A , Heremans JF , Dessy F . Transplantable immunoglobulin‐secreting tumours in rats. IV. Sixty‐three IgE‐secreting immunocytoma tumours. Immunology. 1974;26:713‐723. 4212482 PMC1423177

[23] Miotti S , Canevari S , Menard S , et al. Characterization of human ovarian carcinoma‐associated antigens defined by novel monoclonal antibodies with tumor‐restricted specificity. Int J Cancer. 1987;39:297‐303. 2434438 10.1002/ijc.2910390306

[24] Charles River Laboratories . Baseline haematology and clinical chemistry values for Charles River Wistar rats as a function of sex and age. 1998.

[25] Karagiannis SN , Bracher MG , Beavil RL , et al. Role of IgE receptors in IgE antibody‐dependent cytotoxicity and phagocytosis of ovarian tumor cells by human monocytic cells. Cancer Immunol Immunother. 2008;57:247‐263. 17657488 10.1007/s00262-007-0371-7 PMC11030264

[26] Karagiannis P , Singer J , Hunt J , et al. Characterisation of an engineered trastuzumab IgE antibody and effector cell mechanisms targeting HER2/neu‐positive tumour cells. Cancer Immunol Immunother. 2009;58:915‐930. 18941743 10.1007/s00262-008-0607-1 PMC3017872

[27] Gordon JR , Galli SJ . Mast cells as a source of both preformed and immunologically inducible TNF‐alpha/cachectin. Nature. 1990;346:274‐276. 2374592 10.1038/346274a0

[28] Basal E , Eghbali‐Fatourechi GZ , Kalli KR , et al. Functional folate receptor alpha is elevated in the blood of ovarian cancer patients. PLoS One. 2009;4:e6292. 19617914 10.1371/journal.pone.0006292 PMC2707611

[29] Fazekas‐Singer J , Berroteran‐Infante N , Rami‐Mark C , et al. Development of a radiolabeled caninized anti‐EGFR antibody for comparative oncology trials. Oncotarget. 2017;8:83128‐83141. 29137329 10.18632/oncotarget.20914 PMC5669955

