# Supplementary material for: An immunologically relevant rodent model demonstrates safety of therapy using a tumour‐specific IgE
Source: Allergy. 2018 Oct 8;73(12):2328–41. doi: 10.1111/all.13455 (PMC6492130; doi:10.1111/all.13455)
Supplement: Supplementary file 1 [file ALL-73-2328-s001.docx]

**Supplementary Table 1**

**A**

|  | **Severity of Clinical Sign** | | |
| --- | --- | --- | --- |
| **Clinical Sign Category** | **Mild** | **Moderate** | **Severe** |
| Food and water consumption | 40 – 75% of normal for 72 hours | < 40% of normal for 72 hours | < 40% of normal for 7 days |
| Piloerection | Partial piloerection | Marked piloerection (staring coat) | Marked piloerection (staring coat) with other signs of dehydration |
| Responsiveness | Subdued but responsive, normal provoked patterns of behaviour | Subdued with subdued behaviour patterns even when provoked | Unresponsive to extraneous activity and provocation |
| Peer interaction | Interacts with peers | Little peer interaction |  |
| Hunching | Hunched transiently especially after dosing | Hunched intermittently | Hunched persistently (‘frozen’) |
| Vocalisation | Transient vocalisation | Intermittent – vocalisation when provoked | ‘Distressed’ – vocalisation unprovoked |
| Oculo-nasal discharge | Transient | Persistent | Persistent and copious |
| Respiration | Normal respiration | Intermittent abnormal breathing pattern | Laboured respiration |
| Tremors | Transient | Intermittent | Persistent |
| Convulsions | None | Intermittent | Persistent |
| Prostration | None | Transient prostration (< 1 hour) | Prolonged prostration (> 1 hour) |
| Self-mutilation | None | None | Self-mutilation |

**B**

| **Score** | **Symptoms** |
| --- | --- |
| 0 | No symptoms |
| 1 | Scratching and rubbing around nose and head |
| 2 | Swelling around eyes and mouth; diarrhoea; pilar erecti; reduced activity; increased respiratory rate |
| 3 | Wheeze; laboured respiration; cyanosis around mouth and tail |
| 4 | No activity after stimulation; tremor or convulsions |
| 5 | Death |
