# Supplementary material for: An immunologically relevant rodent model demonstrates safety of therapy using a tumour‐specific IgE
Source: Allergy. 2018 Oct 8;73(12):2328–41. doi: 10.1111/all.13455 (PMC6492130; doi:10.1111/all.13455)
Supplement: Supplementary file 2 [file ALL-73-2328-s002.docx]

**Supplementary Table 2**

|  |  | **PBS** | **Rat MOv18 IgE** | | | **Rat MOv18 IgG** | | |
| --- | --- | --- | --- | --- | --- | --- | --- | --- |
|  |  | **N/A** | **5**  **mg/kg** | **10**  **mg/kg** | **50**  **mg/kg** | **5**  **mg/kg** | **10**  **mg/kg** | **50**  **mg/kg** |
| **No. of animals observed (%)** |  | 32 | 20 | 21 | 12 | 7 | 12 | 1 |
| **Clinical Sign Category/Severity** | **Highest Toxicity Observed** |  |  |  |  |  |  |  |
| Reduced food and water intake | **0** | 32 (100) | 20 (100) | 21 (100) | 12 (100) | 07 (100) | 12 (100) | 1 (100) |
| Piloerection | **0** | 32 (100) | 17 (85) | 20 (95) | 10 (83) | 5 (71) | 9 (75) | 0 (0) |
|  | **1** | 0 (0) | 3 (15) | 1 (5) | 2 (17) | 1 (29) | 3 (25) | 1 (100) |
| Reduced responsiveness | **0** | 10 (31) | 10 (50) | 10 (48) | 9 (75) | 0 (0) | 0 (0) | 0 (0) |
|  | **1** | 22 (69) | 10 (50) | 11 (52) | 3 (25) | 7 (100) | 12 (100) | 1 (100) |
| Reduced peer interaction | **0** | 32 (100) | 20 (100) | 21 (100) | 12 (100) | 7 (100) | 12 (100) | 1 (100) |
| Hunching | **0** | 32 (100) | 14 (70) | 16 (76) | 10 (83) | 3 (43) | 5 (42) | 0 (0) |
|  | **1** | 0 (0) | 6 (30) | 5 (24) | 2 (17) | 4 (57) | 7 (58) | 1 (100) |
| Vocalisation | **0** | 32 (100) | 20 (100) | 21 (100) | 12 (100) | 7 (100) | 12 (100) | 1 (100) |
| Oculo-nasal discharge | **0** | 32 (100) | 20 (100) | 21 (100) | 12 (100) | 7 (100) | 12 (100) | 1 (100) |
| Laboured respiration | **0** | 32 (100) | 20 (100) | 21 (100) | 12 (100) | 7 (100) | 12 (100) | 1 (100) |
| Tremors | **0** | 32 (100) | 20 (100) | 21 (100) | 12 (100) | 7 (100) | 12 (100) | 1 (100) |
| Convulsions | **0** | 32 (100) | 20 (100) | 21 (100) | 12 (100) | 7 (100) | 12 (100) | 1 (100) |
| Prostration | **0** | 32 (100) | 20 (100) | 21 (100) | 12 (100) | 7 (100) | 12 (100) | 1 (100) |
| Self-mutilation | **0** | 32 (100) | 20 (100) | 21 (100) | 12 (100) | 7 (100) | 12 (100) | 1 (100) |
|  | **Toxicity severities were scored as:** **0 = no toxicity, 1 = mild, 2 = moderate, 3 = severe** | | | | | | | |

**Supplementary Table 2 General toxicities observed following rat MOv18 antibody administration.** Numbers outside parentheses represent the number of animals per group that experienced the specific toxicity at a particular severity. Numbers inside parentheses represent the percentage of animals per group to experience the toxicity.
