# Supplementary material for: An immunologically relevant rodent model demonstrates safety of therapy using a tumour‐specific IgE
Source: Allergy. 2018 Oct 8;73(12):2328–41. doi: 10.1111/all.13455 (PMC6492130; doi:10.1111/all.13455)
Supplement: Supplementary file 4 [file ALL-73-2328-s004.docx]

**Supporting Information**

**Supplementary Materials and Methods**

**Cell lines and culture**

The CC531tFR cell line, originally derived from a 1,2 dimethyhydrazine (DMH)-induced colon adenocarcinoma of a WAG-Rij rat ([1](#_ENREF_1)) (Cell Lines Service), was transfected to express the human tumor-associated antigen folate receptor alpha (FRα) and selected on the basis of Geneticin resistance as previously-described (S. Canevari, M. Colnaghi, Instituto Nazionale Tumori, Milan) ([2](#_ENREF_2), [3](#_ENREF_3)). IGROV1 human ovarian carcinoma cells naturally over-express human FRα ([4](#_ENREF_4), [5](#_ENREF_5)). A375 (human metastatic melanoma; CRL-11147) and RBL-2H3 (Wistar rat basophilic leukaemia; CRL-2256) cells were from ATCC. FreeStyle^TM^ 293-F cells (R790-07), a derivative of the human embryonic kidney cell line (HEK293), were from Invitrogen. A-375 cells were maintained in DMEM containing 10% fetal calf serum (FCS). RBL-2H3, CC531tFR and IGROV1 cells were maintained in RMPI-1640 containing 10% FCS and 1.2 mg/mL Geneticin G418 (Gibco) supplementation for CC531tFR cells. FreeStyle^TM^ 293-F cells were maintained in FreeStyle™ 293 Expression Medium (Invitrogen) under serum-free conditions. Cells were grown in 2 mM L-glutamine, penicillin (5,000 U/mL), and streptomycin (100 μg/mL). All cells were maintained in a 5% CO2 humidified incubator at 37°C.

**Isolation of rat immune effector cells from peripheral blood**

Rat primary monocytes were prepared from rat peripheral blood leukocytes (PBL) by flow cytometry cell sorting using a PE-conjugated antibody recognizing CD172 (BD Biosciences) and used freshly for antibody characterization.

**Flow cytometric evaluations of human and rat antibody binding to cell surface receptors**

For assessment of rMOv18 IgE or IgG2b antibody binding to FRα on FRα-expressing tumor cell lines (IGROV1 and CC531tFR), a non-FRα-expressing tumor cell line (A375), rat primary monocytes, or RBL-2H3 cells, cells were incubated with 10 μg/mL mAbs for 30 minutes at 4°C, followed by 2 washes in PBS with 5% normal goat serum (FACS buffer). Cells were then treated with mouse anti-rat IgE-FITC (Pierce) or goat anti-rat IgG2b-FITC (AbD Serotec) for 30 minutes at 4°C and washed in FACS buffer prior to acquisition and analysis on a FACS Canto flow cytometer (BD Biosciences).

**Quantitative analysis of gene expression by antibody-treated tumor-bearing rat lungs**

Tumor-bearing rat lungs were suspended in buffer RLT supplemented with β-mercaptoethanol and stored at -20°C until RNA purification. Total RNA was prepared from tumour-bearing rat lungs using the RNeasy Plus Mini Kit (Qiagen) according to the manufacturer’s protocol. Lungs were studied for transcriptome profiling using the GeneChip™ Rat Gene 1.0 ST Array (ThermoFisher Scientific) and Qlucore software, and subject to microarray pre-processing and Gene Set Enrichment Analysis.

**Microarray pre-processing and Gene Set Enrichment Analysis**

CEL files were read into R using the oligo package ([6](#_ENREF_6)), normalised and log2 transformed with the Robust Multi-chip Average method (RMA) ([7](#_ENREF_7)). Genes were annotated based upon the NetAffx annotations and those without an assigned gene symbol were removed from the dataset. For transcript clusters assigned to multiple gene symbols, a representative was selected using the “MaxMean” parameter in the collapseRows package ([8](#_ENREF_8)) resulting in 19211 genes.

Gene Set Enrichment Analysis (GSEA) ([9](#_ENREF_9)) was performed to identify enriched pathways between samples. The analysis was performed on gene sets derived from the BioCarta pathways provided by the Molecular Signatures Database (MsigDB). GSEA was performed with the following parameters. The ranking metric set to “diff of classes”, the enrichment statistic parameter set to “classic”, and the permutation type set to “gene set” with 10000 permutations. Pathways were considered significant if they had an FDR < 0.05.

**Multi-cytokine bead immunoassays**

Serum was collected from whole rat blood on day 30 following tumor challenge. Samples were assayed using the Milliplex® MAP Rat Cytokine/Chemokine Kit (Millipore, Billerica, MA), and acquired on a FLEXMAP 3D analyzer using xPONENT® software (Luminex Corporation), following the manufacturer’s instructions (analytes in the panel: TNFα, MCP-1, IFN-γ, RANTES, IL-4, IL-5, IL-6, IL-10, IL-12, IL-13). Cytokine concentrations were calculated from the standard curve.

**Rat anti-chimeric antibody ELISA**

Rat anti-chimeric antibody concentrations were measured in sera collected from whole rat blood on day 30 following tumor challenge, using an in-house developed ELISA. Briefly, 96-well plates were coated overnight at 4°C with 2 μg/mL rat MOv18 IgE in carbonate-bicarbonate buffer. After blocking buffer incubation (SuperBlock buffer, 0.05% Tween-20; Perbio Science UK Ltd), plates were washed and incubated with mouse anti-rat IgE heavy chain antibody (Bio-Rad) or rat sera (1:5 in blocking buffer) for 2.5 h at room temperature. Plates were again washed and incubated with 40 ng/mL peroxidase-AffiniPure F(ab')2 Fragment Goat Anti-Rat IgG (Stratech Scientific) in blocking buffer for 1 h at room temperature. Following five washes, samples were treated with 0.5 mg/mL o-phenylenediamine dihydrochloride substrate (Sigma-Aldrich) in peroxide substrate buffer (Pierce) followed by stop solution (1M HCl). Optical density values were measured at 492 nm (reference wavelength: 650 nm). Standard curve fitting was performed using Graphpad Prism software (Graphpad) with a 4-parameter curve fit using a minimum of 6 points on the standard curve.

**Rat anti-human FRα antibody ELISA**

Rat anti-human FRα antibody concentrations were measured in sera collected from whole rat blood on day 30 following tumor challenge, using an in-house developed ELISA. Briefly, 96-well plates were coated overnight at 4°C with 1 μg/mL recombinant human FRα (R&D Systems) in carbonate-bicarbonate buffer. After blocking buffer incubation (SuperBlock buffer, 0.05% Tween-20; Perbio Science UK Ltd), plates were washed and incubated with rat MOv18 IgG2b antibody or rat sera (1:5 in blocking buffer) for 2.5 h at room temperature. Plates were again washed and incubated with 45 ng/mL Peroxidase-AffiniPure F(ab')2 Fragment Donkey Anti-Rat IgG (H+L) (Stratech Scientific) in blocking buffer for 1 h at room temperature. Following five washes, samples were treated with 0.5 mg/mL o-phenylenediamine dihydrochloride substrate (Sigma-Aldrich) in peroxide substrate buffer (Pierce) followed by stop solution (1M HCl). Optical density values were measured at 492 nm (reference wavelength: 650 nm). Standard curve fitting was performed using Graphpad Prism software (Graphpad) with a 4-parameter curve fit using a minimum of 6 points on the standard curve

**Supplementary References**

1. Marquet RL, Westbroek DL, Jeekel J. Interferon treatment of a transplantable rat colon adenocarcinoma: importance of tumor site. International journal of cancer. Journal international du cancer 1984;33(5):689-92.

2. Coney LR, Tomassetti A, Carayannopoulos L, Frasca V, Kamen BA, Colnaghi MI, et al. Cloning of a tumor-associated antigen: MOv18 and MOv19 antibodies recognize a folate-binding protein. Cancer research 1991;51(22):6125-32.

3. Bottero F, Tomassetti A, Canevari S, Miotti S, Menard S, Colnaghi MI. Gene transfection and expression of the ovarian carcinoma marker folate binding protein on NIH/3T3 cells increases cell growth in vitro and in vivo. Cancer research 1993;53(23):5791-6.

4. Gould HJ, Mackay GA, Karagiannis SN, O'Toole CM, Marsh PJ, Daniel BE, et al. Comparison of IgE and IgG antibody-dependent cytotoxicity in vitro and in a SCID mouse xenograft model of ovarian carcinoma. European journal of immunology 1999;29(11):3527-37.

5. Benard J, Da Silva J, De Blois MC, Boyer P, Duvillard P, Chiric E, et al. Characterization of a human ovarian adenocarcinoma line, IGROV1, in tissue culture and in nude mice. Cancer research 1985;45(10):4970-9.

6. Carvalho BS, Irizarry RA. A framework for oligonucleotide microarray preprocessing. Bioinformatics 2010;26(19):2363-7.

7. Irizarry RA, Hobbs B, Collin F, Beazer-Barclay YD, Antonellis KJ, Scherf U, et al. Exploration, normalization, and summaries of high density oligonucleotide array probe level data. Biostatistics 2003;4(2):249-64.

8. Miller JA, Cai C, Langfelder P, Geschwind DH, Kurian SM, Salomon DR, et al. Strategies for aggregating gene expression data: the collapseRows R function. BMC bioinformatics 2011;12:322.

9. Subramanian A, Tamayo P, Mootha VK, Mukherjee S, Ebert BL, Gillette MA, et al. Gene set enrichment analysis: a knowledge-based approach for interpreting genome-wide expression profiles. Proceedings of the National Academy of Sciences of the United States of America 2005;102(43):15545-50.
